# Healthcare use by 30,000 patients with irritable bowel syndrome (IBS) in France: a 5-year retrospective and one-year prospective national observational study

**DOI:** 10.1186/s12876-019-1031-z

**Published:** 2019-06-27

**Authors:** Jean-Marc Sabaté, Sébastien Rivière, Pauline Jouet, Christelle Gastaldi-Menager, Anne Fagot-Campagna, Philippe Tuppin

**Affiliations:** 1 Service de Gastroentérologie, Hôpital Avicenne AP-HP, INSERM U-987, 175 rue de Stalingrad, 93000 Bobigny, France; 2 Physiopathologie et Pharmacologie Clinique de la Douleur, Hôpital Ambroise Paré, 9 avenue Charles-de-Gaulle, 92104 Boulogne-Billancourt, France; 3 Caisse nationale de l’Assurance maladie (CNAM) - Direction de la Stratégie des Études et des Statistiques, 26-50 avenue du Professeur André Lemierre, F-75986 Paris, Cedex 20 France; 4 Service de Gastroentérologie, Hôpital Ambroise Paré AP-HP, 9 avenue Charles-de-Gaulle, 92104 Boulogne-Billancourt, France

**Keywords:** Irritable bowel syndrome, Comorbidities, Hospitalisation, Ambulatory healthcare use, Observational study

## Abstract

**Background:**

Irritable bowel syndrome (IBS) can be responsible for alteration in quality of life and economic burden. The aim of this study was to evaluate healthcare use related to this disorder in France.

**Methods:**

The French health data system was used to select adults covered by the general health scheme (87% of population) through their first IBS hospitalization in 2015. We studied the healthcare refunded during the previous 5 years, 1 year before and after hospitalization.

**Results:**

Among 43.7 million adults who used refunded healthcare in 2015, 29,509 patients were identified (0.07, 33% males, 67% females, mean age 52 years, 30% admitted through emergency room). During their hospitalization, 33% had upper endoscopy and 64% colonoscopy. Over the five previous years, 3% had at least one hospitalization with an IBS diagnosis, 58% had abdominal ultrasonography, 27% CT scan, 21% upper endoscopy, 13% colonoscopy and 83% a gastroenterologist visit. The year before, these rates were respectively: 0, 36, 16, 6, 4 and 78%. Some of those rates decreased the year after the hospitalization with respectively: 1, 27, 13, 5, 4 and 19%. The year before, 65% had at least one CRP dosage (13% three or more), 58% a TSH dosage (7%) and 8% a test for coeliac diseases (1%) and the year after: 44% (8%), 43% (5%) and 3% (0.3%). At least one refund of a drug used to treat IBS was found for 85% of patients 5 years before, 65% one year before and 51% one year after.

**Conclusion:**

This first study using French health data system for healthcare consumption assessment in IBS points out the repetition of outpatient visits, examinations and in particular radiological examinations, without a strong decrease after hospitalization for IBS and gastroenterologist visit.

## Background

Irritable bowel syndrome (IBS) is a chronic functional gastrointestinal (GI) disorder characterised by abdominal pain and altered bowel habit [1, 2] The prevalence of IBS in adults is high, estimated by a meta-analysis to be 12% in North America, 21% in South America and 7% in Southeast Asia [3–6]. In France, a recent web-based survey reported a prevalence of IBS of 10% of adults [6, 7]. Various subtypes have been described based on the predominant stool pattern, including IBS with constipation (IBS-C), IBS with diarrhoea (IBS-D), and IBS with mixed bowel patterns of both constipation and diarrhoea (IBS-M) according to the new Rome IV criteria [1].

While IBS is considered to be a benign disease usually not associated with any excess mortality, it can have an impact on quality of life and healthcare use, which can be explained by various factors [8–15]. First, the pathophysiology of IBS is complex involving multiple peripheral and central mechanisms that explain the globally limited efficacy of treatments in this heterogeneous disease [2]. Secondly, no diagnostic test is currently available and all examinations performed in the context of IBS are designed to eliminate other diagnoses [2]. Taken together, all of these factors can explain the unfulfilled expectations of many patients, who repeatedly consult various physicians (medical nomadism) and try various treatments in an attempt to improve their condition [10, 11]. The severity of the disease varies from patient to patient, but about 20–25% of patients have severe IBS and these patients account for the maximum use of healthcare resources. Treatment failure also has an economic impact [4, 9, 16, 17]. These patients therefore present high consumption of healthcare resources, and the economic burden of direct and indirect costs has been extensively studied and has been estimated to be similar to that of other chronic diseases such as hypertension in the USA [16–19]. In France, the most recent data are derived from a 2003 study, in which the average cost was about €750 per patient per year with an average of 3.2 days of sick leave [7].

Studies on healthcare use of patients with IBS and their costs have mainly been based on population samples, practitioners, existing cohort studies or claims data in outpatient groups selected by ICD 9 codes or drugs indicated for IBS. Determining the nationwide characteristics of IBS patients and their healthcare utilisation constitutes an important step towards a better understanding of the public health impact of this disease and how IBS patients are treated.

We conducted a nationwide observational population-based study using the French National Health data System (*Système National des Données de Santé* SNDS) to estimate the utilisation of healthcare resources 5 years before and one year after hospitalisation with a diagnosis of IBS in 2015.

## Methods

### Data sources and population

The SNDS database comprehensively and individually records all outpatient prescriptions and healthcare procedures reimbursed to beneficiaries of the various French health schemes [20]. An anonymous and unique identification number for each beneficiary allows this information to be linked to the data collected by the National hospital discharge database (*Programme de médicalisation des systèmes d’information PMSI*) during hospital stays in the various types of public and private healthcare institutions in France. Hospital diagnoses are coded according to the International Classification of Diseases 10th edition (ICD-10), in the same way as the diagnoses allowing attribution of LTD status. Attribution of long-term disease (LTD) status for a severe and costly chronic disease is also recorded. LTD status is validated by a national health insurance physician at the request of the attending physician, allowing exemption of co-payment, and can provide information about the nature of the diseases treated. Medical procedures performed on an outpatient basis or in hospital are identified by the *Classification Commune des Actes Médicaux* [common classification of medical procedures]. Upper GI endoscopy and colonoscopy, outpatient and inpatient abdominal ultrasound, CT scan or MRI can also be identified. Outpatient reimbursed drugs are identified by their ATC code (Anatomical Therapeutic Classification) and their therapeutic class, while outpatient laboratory procedures are identified by the *Nomenclature des Actes de Biologie Médicale* [clinical pathology test nomenclature]. Medical visits are identified by the *Nomenclature Générale des Actes Professionnels* [general nomenclature of professional procedures]. Physician visits include private practice visits and hospital outpatient department visits, but not impatient care.

The national health insurance general health scheme and local mutualist sections (students, civil servants, etc) covered about 87% of the 66 million inhabitants of France in 2015. The *Mutualité Sociale Agricole* (agricultural workers’ health insurance fund) and the *Régime Social des Indépendants* (self-employed health insurance fund) each cover 5% of the population, and the remaining 4% are covered by other schemes. The population of the present study was therefore composed of general health scheme beneficiaries (18 years or over), including local mutualist sections (58 million inhabitants) with at least one annual healthcare reimbursement (for any form of healthcare) per year during the study period.

Because of the possible similarity of symptoms, people with inflammatory bowel disease and/or colon cancer were excluded by using their LTD ICD-10 codes or hospitalisation diagnosis during the study period. IBS cases in 2015 were selected using hospital discharge principal diagnoses to identify individuals discharged from hospital at least once: ICD-10 codes K58.0: Irritable bowel syndrome with diarrhoea and K58.9: Irritable bowel syndrome without diarrhoea.

### Data analysis

The presence of the intestinal and extra-intestinal comorbidities commonly associated with IBS were investigated by using hospital principal diagnosis. Healthcare procedures, examinations, visits, medication, and hospitalisation rates were calculated by including people with at least one reimbursement during 2015, and during the 5 years before or the year after hospitalisation. Data are expressed as mean ± standard deviation (SD). Reimbursement rates (0, 1, 2, 3 or more or one or more) one year before and one year after hospitalisation were compared using McNemar’s test for paired nominal data.

All analyses were performed with SAS Enterprise Guide software (version 7.1, SAS Institute Inc., Cary, NC, USA). Specific ethics committee approval was not required for this study. The CNAM (*Caisse nationale d’assurance maladie*) has permanent access to the SNDS database approved by decree and the French data protection authority (*Commission Nationale de l’Informatique et des Libertés).*

## Results

### Patient characteristics

**Fig. 1:**
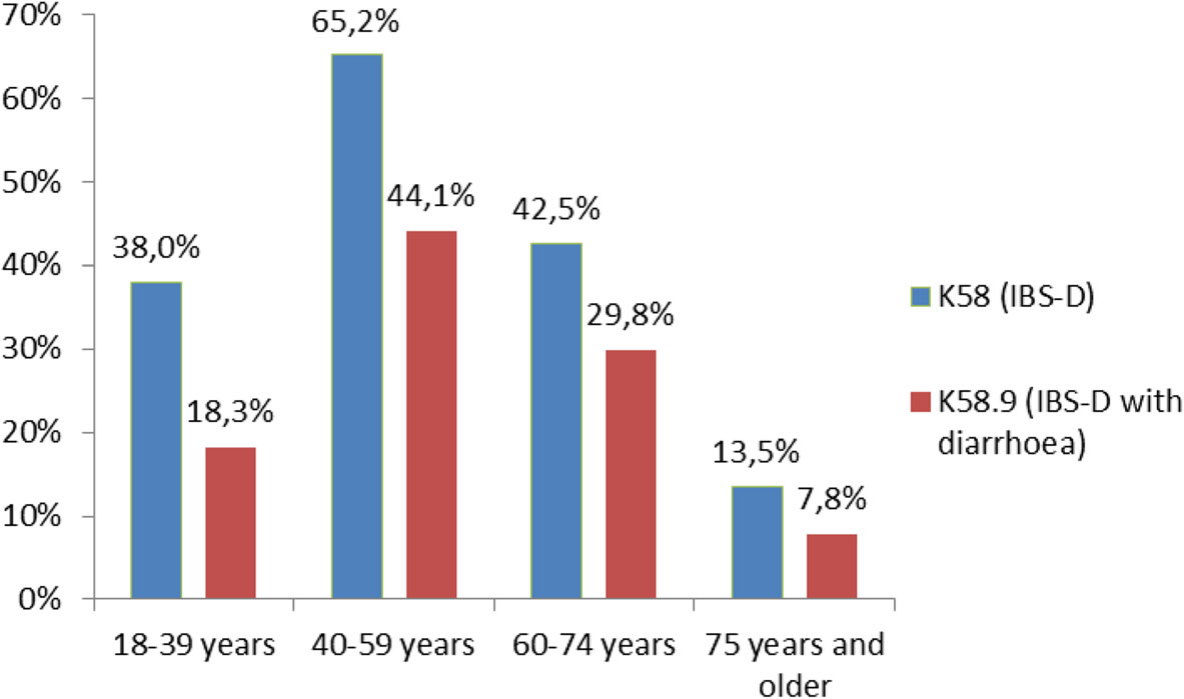
Age distribution of the two IBS ICD-10 diagnoses in France in 2015

The study was based on 43,675,462 adults (44.9% males and 55.1% females), including 29,509 patients with at least one hospital principal diagnosis ICD-10 code for IBS in 2015: 9821 males (33%) and 1688 females (67%); 0.07, 0.05 and 0.08% of the initial population, respectively. A specific IBS-D ICD-10 diagnosis code was identified for 10,995 (37.3%) of these patients. Sixty-five per cent of patients with a K58.0 code and 68% of patients with a K58.9 code were female. The mean age of all IBS patients was 52.1 years (SD ±16.3 (men: 51.5 years SD ±15.9 and women 52.4 years SD ±16.5), 10.7% were younger than 30 years and 8.5% of patients were 75 years or older. Patients with IBS-D were younger than patients with IBS without diarrhoea (49.5 years: SD ±18.0 vs 53.7 years SD ±15.1); the age distribution of IBS ICD-10 diagnosis by gender is shown in Fig. 1.

### Inpatient admission

Admission via an emergency room was identified for 30% of the patients included (40% for patients aged 18–39 years and 44% for patients 75 years or older) (Table 1). The overall mean length of hospital stay (LOS) in 2015 was 1.4 days (SD ±1.97), and was highest for people 75 years or older and the mean number of hospital stays for a diagnosis of IBS in 2015 was 1.03 (SD ±0.18). Over the five years before inclusion in 2015, 3% of patients were hospitalised at least once for IBS, 0.3% were hospitalised at least once during the year before the index hospitalisation with a significant increase to 1.1% during the year after the index hospitalisation for all age groups (Table 2). All-diagnosis hospitalisation rates were 65% five years before the index hospitalisation, 26% one year before the index hospitalisation and 27% during the year after the index hospitalisation, also corresponding to a significant increase. The most common GI hospital diagnoses other than IBS during the five years before the index hospitalisation were abdominal and pelvic pain (5%), gastritis and duodenitis (4%), disorders of gallbladder and biliary tract (3%), and gastroesophageal reflux disease (2%). As if was not the case for IBS hospitalisation diagnosis, most of these hospitalisation rates decreased significantly one year after hospitalisation of IBS compared to one year before.

**Table 1:** Admission via the emergency room, length of hospital stay selected for inclusion and number of hospital stays in 2015 with a principal diagnosis of IBS by age and IBS code

Age (years)	18–39	40–59	60–74	75 or older	Total
Admission via emergency room (%)					
k58.0	37.8	29.3	27.0	52.2	33.9
k58.9	42.0	25.6	22.3	37.8	28.5
k58.0 or k58.9	39.7	26.8	23.7	43.9	30.5
Length of hospital stay					
K58.0 (mean SD)	1.21 (±1.01)	1.32 (±1.39)	1.70 (±2.20)	4.59 (±6.15)	1.68 (±2.58)
k58.9 (mean SD)	1.19 (±0.82)	1.16 (±0.85)	1.28 (±1.18)	2.44 (±3.91)	1.30 (±1.47)
K58.0 or K58.9 (mean SD)	1.20 (±0.92)	1.21 (±1.06)	1.40 (±1.56)	3.36 (±5.10)	1.44 (±1.97)
Hospital stays					
K58.0 (mean SD)	1.04 (±0.20)	1.04 (±0.20)	1.02 (±0.15)	1.01 (±0.12)	1.03 (±0.19)
k58.9 (mean SD)	1.05 (±0.21)	1.03 (±0.17)	1.01 (±0.13)	1.01 (±0.09)	1.03 (±0.16)
K58.0 or K58.9 (mean SD)	1.04 (±0.21)	1.03 (±0.19)	1.02 (±0.14)	1.01 (±0.11)	1.03 (±0.18)

K58.0 Irritable bowel syndrome with diarrhoea, K58.9 Irritable bowel syndrome without diarrhoea

**Table 2:** At least one short-stay hospitalisation five years to one year before or one year after hospitalisation with a diagnosis of IBS in France in 2015 according to the most common diagnoses

	Before hospitalisation									After hospitalisation					
Years	-5	−4	−3	−2	−1										
Age	All	All	All	All	All^a^	18–39	40–59	60–74	75 +	All^a^	P^a^	18–39	40–59	60–74	75 +
	%	%	%	%	%	%	%	%	%	%	%	%	%	%	%
All diagnoses															
1 or more	64.7	57.9	49.6	39.3	25.5	23.9	21.9	26.9	42.5	26.6	< 0.0001	22.6	23.2	29.3	45.5
K58.0 or K58.9															
1 or more	3.1	2.0	1.2	0.6	0.3	0.3	0.2	0.3	0.4	1.1	< 0.0001	1.4	0.9	1.1	1.4
Abdominal or pelvic pain (R10)															
1 or more	4.8	4.1	3.4	2.6	2.0	2.9	1.6	1.4	2.8	1.2	< 0.0001	1.8	0.9	0.8	2.0
Gastric diseases (K25-K29)															
1 or more	4.6	3.8	3.0	2.2	1.3	1.4	1.3	1.1	1.2	0.8	< 0.0001	0.8	0.7	0.9	0.6
Gastritis and duodenitis (K29)															
1 or more	4.3	3.5	2.8	2.0	1.1	1.2	1.2	1.1	0.9	0.7	< 0.0001	0.8	0.7	0.7	0.4
Hernias (K40–46)															
1 or more	3.3	2.8	2.2	1.6	0.9	0.5	0.8	1.2	1.2	1.1	< 0.0001	0.5	1.1	1.4	1.4
Diaphragmatic hernia (K44)															
1 or more	0.8	0.7	0.5	0.4	0.3	0.2	0.2	0.4	0.4	0.1	0.0048	0.1	0.1	0.2	0.2
Gallbladder and biliary tract disorders (K80–83)															
1 or more	3.2	2.8	2.2	1.6	0.9	0.8	0.9	1.0	1.1	1.0	< 0.0001	0.8	1.0	1.1	1.4
Benign neoplasm of colon, rectum, anus and anal canal (D12)															
1 or more	2.8	2.1	1.1	0.5	0.2	0.1	0.2	0.4	0.4	0.3	0.6396	0.1	0.3	0.5	0.8
Diseases of oesophagus (K20–23)															
1 or more	2.6	2.1	1.6	1.1	0.5	0.5	0.6	0.5	0.5	0.4	< 0.0001	0.4	0.5	0.3	0.4
Gastro-oesophageal reflux disease (K21)															
1 or more	1.9	1.5	1.2	0.8	0.4	0.4	0.5	0.4	0.4	0.2	0.0002	0.2	0.2	0.1	0.0
Other functional intestinal disorders (K59)															
1 or more	2.3	1.8	1.5	1.1	0.8	0.9	0.7	0.6	2.2	0.6	< 0.0001	0.6	0.4	0.5	1.5
Diverticular disease of intestine (K57)															
1 or more	2.1	1.6	1.2	0.9	0.6	0.3	0.5	0.7	1.6	0.4	< 0.0001	0.2	0.3	0.5	1.4
Diseases of anus and rectum including haemorrhoids (K60–62. 64)															
1 or more	2.0	1.4	1.1	0.8	0.5	0.8	0.5	0.4	0.7	0.7	0.0001	0.8	0.8	0.5	0.8
Intestinal infectious diseases (A00-A09)															
1 or more	1.4	1.2	1.0	0.9	0.7	1.2	0.5	0.4	1.4	0.3	< 0.0001	0.3	0.1	0.2	0.9
Benign neoplasm of other and ill-defined parts of digestive system (D13)															
1 or more	0.4	0.3	0.3	0.2	0.1	0.1	0.1	0.1	0.0	0.1	< 0.0001	0.1	0.1	0.2	0.2
Disease of appendix (K35–38)															
1 or more	0.8	0.7	0.5	0.4	0.2	0.6	0.2	0.1	0.2	0.2	0.0118	0.4	0.1	0.1	0.1
Mood disorders (F30–39)															
1 or more	0.8	0.6	0.5	0.3	0.2	0.1	0.1	0.1	0.8	0.2	< 0.0001	0.1	0.3	0.2	0.4
Neurotic, stress-related and somatoform disorders (F40–48)															
1 or more	0.8	0.6	0.5	0.3	0.2	0.2	0.1	0.2	0.6	0.2	< 0.0001	0.2	0.2	0.3	0.5

^a^P for each distribution comparison (all −1 year vs all 1 year)

### Endoscopic and radiological examinations

The main examinations performed during the hospital stay selected for IBS diagnosis in 2015 were upper GI endoscopy (33%), performed more frequently among younger patients (18–39 years, 41%), and colonoscopy (64%, relatively stable across age groups) comprising polyp removal in 4% of patients (1.7% under the age of 40 years, 3.9% between 40 and 59 years and 5.0% between 60 and 74 years (Table 3). Less than 1% of patients underwent other radiological examination such as ultrasound (0.7%), CT scan (0.4%) or MRI (0%).

**Table 3:** Outpatient endoscopic and radiological examinations five years to one year before hospitalisation, during hospitalisation or one year after hospitalisation of patients with IBS diagnosed in France in 2015 according to the number of reimbursements for certain tests

	Before hospitalisation					During hospitalisation					After hospitalisation				
	−5	−4	−3	−2	− 1										
	All	All	All	All	All	All*	18–39	40–59	60–74	75 +	All*	18–39	40–59	60–74	75 +
Abdominal and pelvic ultrasound															
0	42.0	45.5	50.3	55.6	63.8	99.3	98.9	99.4	99.4	99.3	73.0	70.4	72.9	74.5	76.6
1	31.1	31.6	31.6	31.5	29.2	0.7	1.1	0.6	0.6	0.7	24.2	26.3	24.6	23.0	20.6
2	14.9	13.5	11.7	9.3	5.5	0.0	0.0	0.0	0.0	0.0	2.3	2.8	2.2	2.1	2.2
3 or more	11.9	9.3	6.5	3.6	1.4	0.0	0.0	0.0	0.0	0.0	0.4	0.6	0.3	0.4	0.6
Abdominal X-ray^a^															
0	83.6	86.2	88.5	90.6	93.0	99.7	99.7	99.8	99.6	99.5	94.9	94.4	95.6	95.1	92.3
1	10.4	8.8	7.6	6.5	5.0	0.3	0.3	0.1	0.4	0.5	4.4	5.1	3.8	4.2	6.3
2	3.8	3.2	2.6	2.1	1.5	0.0	0.0	0.0	0.0	0.0	0.5	0.4	0.4	0.5	1.2
3 or more	2.3	1.8	1.3	0.9	0.5	0.0	0.0	0.0	0.0	0.0	0.2	0.1	0.2	0.2	0.2
Abdominal and pelvic CT scan															
0	72.5	74.5	77.1	80.3	84.5	99.6	99.5	99.6	99.6	99.3	87.4	88.0	87.9	87.4	83.4
1	17.4	16.7	15.7	14.3	12.1	0.4	0.5	0.4	0.4	0.7	11.3	10.7	10.9	11.1	14.8
2	6.1	5.6	4.9	3.9	2.7	0.0	0.0	0.0	0.0	0.0	1.1	1.1	1.0	1.2	1.5
3 or more	4.0	3.1	2.4	1.5	0.7	0.0	0.0	0.0	0.0	0.0	0.2	0.2	0.2	0.3	0.4
Abdominal MRI															
0	91.8	92.6	93.5	94.7	96.2	100.0	100.0	100.0	100.0	100.0	96.5	95.3	96.7	97.0	98.0
1	5.7	5.2	4.6	3.8	2.9	0.0	0.0	0.0	0.0	0.0	3.2	4.2	3.1	2.8	1.8
2	1.7	1.6	1.4	1.1	0.8	0.0	0.0	0.0	0.0	0.0	0.2	0.4	0.2	0.2	0.2
3 or more	0.8	0.7	0.5	0.3	0.1	0.0	0.0	0.0	0.0	0.0	0.0	0.1	0.0	0.1	0.0
Upper GI endoscopy															
0	79.0	82.3	85.8	89.2	92.8	67.0	58.9	66.8	69.8	81.9	94.9	95.8	95.7	94.2	91.1
1	15.9	13.9	11.5	9.1	6.4	33.0	41.1	33.1	30.2	18.1	4.9	4.0	4.1	5.7	8.6
2	3.7	2.9	2.1	1.4	0.8	0.1	0.1	0.0	0.0	0.0	0.2	0.2	0.2	0.2	0.3
3 or more	1.4	1.0	0.6	0.3	0.1	0.0	0.0	0.0	0.0	0.0	0.0	0.0	0.0	0.0	0.0
Colonoscopy															
0	87.2	90.6	92.9	94.6	95.6	35.7	33.8	31.4	35.9	60.4	96.2	98.2	97.4	95.1	88.3
1	11.4	8.5	6.5	5.1	4.2	64.3	66.1	68.5	64.0	39.4	3.7	1.7	2.6	4.8	11.6
2	1.2	0.8	0.5	0.3	0.2	0.0	0.1	0.0	0.1	0.1	0.1	0.1	0.0	0.1	0.1
3 or more	0.2	0.1	0.1	0.0	0.0	0.0	0.0	0.0	0.0	0.0	0.0	0.0	0.0	0.0	0.0
Polyp removal during colonoscopy															
0	95.7	96.8	98.0	98.9	99.4	96.3	98.3	96.1	95.0	96.2	99.5	99.8	99.8	99.2	98.5
1	4.0	3.1	1.9	1.1	0.6	3.7	1.7	3.9	5.0	3.8	0.5	0.2	0.2	0.7	1.5
2	0.3	0.2	0.1	0.0	0.0	0.0	0.0	0.0	0.0	0.0	0.0	0.0	0.0	0.0	0.0

^a^with or without contrast agent

*P < 0.0001 for distribution comparisons between “all −1 year” vs “all 1 year”)

During the five years before hospitalisation, 21% of patients had undergone at least one upper GI endoscopy (1% three or more) and 13% had undergone colonoscopy (1% two or more). During the year before and the year after hospitalisation, these rates were 7 and 5% for upper GI endoscopy and 4 and 4% for colonoscopy, respectively. The following examinations were also performed during the year before the diagnosis of IBS: anorectal manometry in 0.2%; defecography in 0.1%; endoscopic ultrasonography in 0.1%. After hospitalisation, these rates were higher for older people (75 years or older: 9 and 12%, respectively). At least one abdominal ultrasound was performed for 58% of people over the five years before, 36% one year before and 27% one year after hospitalisation, while CT scan was performed in 27, 15 and 13% of patients, and MRI was performed in 8, 4 and 3% of patients, respectively. One year before hospitalisation, some patients underwent repeated examinations, such as abdominal ultrasound (12% of patients had undergone 3 or more abdominal ultrasound examinations during the five years before hospitalisation), abdominal and pelvic CT scan (4%), and upper GI endoscopy (1.4%).

### Laboratory tests

The proportion of patients with at least one complete blood count between one year before hospitalisation (86%) decreased significantly compared to one year after hospitalisation (64%) (Table 4). The proportion of patients with CRP also decreased significantly (65% vs 44%). At least one TSH assay was identified for 83% of individuals during the five years before hospitalisation, 58% of patients one year before hospitalisation and 43% of patients one year after hospitalisation. During the five years before hospitalisation, 45% of patients had three or more TSH assays. Serological tests for coeliac disease were performed in 11, 8% (19% for 18–39 years-old) and 3% of patients, respectively. Stools samples for ova and parasites were performed for 15, 10, and 2% of patients, respectively.

**Table 4:** Outpatient laboratory tests five years to one year before, or one year after hospitalisation of patients with IBS diagnosed in France in 2015, according to the number of reimbursements for certain tests

	Before hospitalisation									After hospitalisation				
Years	−5	−4	−3	−2	−1					1 year				
Age	All	All	All	All	All*	18–39	40–59	60–74	75 +	All*	18–39	40–59	60–74	75 +
N					29,509	7040	12,081	7883	2505					
	%	%	%	%	%	%	%	%	%	%	%	%	%	%
CBC and platelet count														
0	2.7	3.4	4.7	7.3	14.3	14.5	16.9	12.0	9.1	36.3	46.6	40.8	26.2	18.2
1	7.0	9.3	13.0	20.1	35.2	35.4	37.3	35.0	25.7	32.5	27.8	32.8	36.7	30.6
2	10.4	13.4	17.6	23.9	25.7	25.9	25.0	27.1	24.4	14.6	12.2	12.9	17.9	19.5
3 or more	79.9	73.9	64.6	48.7	24.7	24.2	20.9	25.9	40.8	16.5	13.4	13.5	19.2	31.7
CRP														
0	13.2	15.6	19.1	24.6	34.5	27.6	37.6	37.1	30.6	55.6	61.0	59.0	50.4	39.7
1	18.7	21.3	24.5	29.0	36.1	39.5	36.8	34.4	28.5	26.6	24.2	26.0	29.0	29.2
2	17.3	18.3	19.7	20.6	16.8	19.5	15.5	16.0	17.5	9.4	8.2	8.3	11.0	13.5
3 or more	50.9	44.8	36.7	25.8	12.6	13.4	10.1	12.5	23.5	8.4	6.6	6.7	9.6	17.7
T.S.H.														
0	17.3	20.0	23.6	29.6	41.7	41.9	43.4	39.8	38.7	56.9	65.9	58.7	49.4	46.5
1	20.4	22.7	26.2	31.2	38.0	40.2	38.2	37.3	32.8	28.7	23.8	28.7	32.5	31.0
2	17.2	18.5	19.7	20.2	13.8	13.0	12.7	15.2	16.5	8.9	6.6	7.9	11.1	13.6
3 or more	45.1	38.8	30.5	19.0	6.6	4.9	5.7	7.7	12.0	5.4	3.7	4.6	7.1	8.9
Tests for coeliac disease														
0	89.4*	89.7	90.2	90.8	91.8	81.5	93.2	96.7	98.4	97.0	94.6	97.4	97.9	99.0
1	6.2	6.1	5.9	5.6	5.2	11.6	4.3	2.1	0.9	1.8	3.4	1.5	1.3	0.5
2	3.2	3.1	2.9	2.7	2.4	5.4	2.0	0.9	0.6	0.8	1.4	0.8	0.5	0.4
3 or more	1.2	1.1	1.0	0.8	0.6	1.5	0.5	0.2	0.2	0.3	0.7	0.2	0.2	0.2
Stool examination for ova														
0	85.3	86.3	87.3	88.5	90.0	83.0	91.5	93.1	92.7	97.5	96.2	97.9	97.8	98.0
1	10.6	10.0	9.4	8.6	7.7	13.3	6.5	5.0	6.3	1.8	2.5	1.5	1.7	1.8
2	1.7	1.5	1.3	1.1	0.8	1.4	0.7	0.6	0.4	0.3	0.5	0.2	0.2	0.1
3 or more	2.4	2.2	2.0	1.8	1.5	2.3	1.4	1.3	0.7	0.5	0.8	0.4	0.4	0.1

*P < 0.0001 for distribution comparisons between “all −1 year” vs “all 1 year”)

### Medications

At least one reimbursement for drugs used to treat functional GI disorders was identified for 85% of patients 5 years before hospitalisation, 65% of patients one year before hospitalisation and 51% of patients one year after hospitalisation (Table 5). The decreased rate of reimbursement for these drugs between one year before hospitalisation and after hospitalisation concerned all age groups, but mainly concerned the “drugs for functional disorders” subgroup. Laxatives were commonly used (one reimbursement was identified for 95% of patients during the five years before hospitalisation, 92% of patients one year before hospitalisation and 89% of patients one year after hospitalisation). The most commonly used subclass was “osmotically acting laxatives”. The most marked decrease in drug use was observed for “Antidiarrheals, intestinal antiinflammatory/antiinfective agents” with 49, 25, and 18% of individuals with at least one reimbursement, respectively.

**Table 5:** Treatments five to one year before or one year after hospitalisation with a diagnosis of IBS in France in 2015 according to ATC code and the number of reimbursements of some drugs

	Before hospitalisation									After hospitalisation				
Year	−5	−4	−3	−2	−1 year					+ 1 year				
Age	All	All	All	All	All*	18–39	40–59	60–74	75 +	All*	18–39	40–59	60–74	75 +
Drugs (ATC code)	%	%	%	%	%	%	%	%	%	%	%	%	%	%
Drugs for functional GI disorders (A03)														
0 reimbursement	14.7	17.5	21.2	26.5	34.8	27.0	35.4	40.4	36.9	48.9	42.5	49.5	53.9	49.0
1–2	24.0	26.4	29.0	32.4	35.4	39.5	36.8	31.6	29.1	33.1	37.7	33.7	29.3	28.8
3 or more	61.3	56.1	49.8	41.1	29.7	33.5	27.8	28.0	34.1	18.0	19.8	16.8	16.8	22.2
Drugs for functional GI disorders (A03A)														
0	19.7	22.8	26.8	32.3	40.7	32.1	41.0	47.0	44.4	53.9	47.3	54.1	59.1	55.0
1–2	29.2	31.0	32.8	34.8	36.0	42.8	37.0	30.8	28.5	32.3	38.2	32.9	27.7	27.3
3 or more	51.1	46.2	40.5	32.9	23.2	25.1	22.0	22.2	27.1	13.8	14.5	13.0	13.1	17.7
Antispasmodics or anticholinergics in combination with other drugs (A03C-D-E)														
0	80.2	82.0	84.1	86.2	89.1	88.7	89.0	89.2	89.8	92.4	92.3	92.3	92.7	92.6
1–2	15.3	14.2	12.9	11.5	9.5	10.4	9.8	8.9	7.8	6.8	7.3	7.1	6.3	5.9
3 or more	4.6	3.8	3.1	2.2	1.4	0.9	1.2	1.8	2.4	0.7	0.4	0.6	0.9	1.6
Propulsives (A03F)														
0	54.9	61.0	67.2	74.7	82.8	76.9	84.3	85.7	82.4	89.8	86.7	90.8	91.2	89.1
1–2	30.6	28.2	25.0	20.4	15.0	20.8	14.0	11.9	13.0	9.3	12.4	8.6	7.8	9.1
3 or more	14.5	10.9	7.8	4.9	2.3	2.2	1.7	2.4	4.6	0.9	0.8	0.6	1.1	1.8
Laxatives (A06)														
0	4.8	5.2	5.8	6.7	7.8	9.4	5.5	6.4	19.4	11.3	12.4	8.8	9.8	24.7
1–2	57.9	61.3	64.7	68.3	72.6	75.5	76.7	72.0	46.0	74.9	77.1	78.7	74.9	50.8
3 or more	37.3	33.5	29.5	25.0	19.6	15.1	17.8	21.6	34.5	13.8	10.5	12.5	15.3	24.5
Laxatives (A06)^a^														
0	33.7	35.9	38.6	41.8	46.2	48.2	46.9	45.4	39.8	52.0	53.6	52.6	51.0	47.6
1–2	38.2	38.7	39.2	39.6	39.9	42.5	41.2	38.8	30.4	38.9	40.3	39.5	38.5	33.0
3 or more	28.1	25.3	22.1	18.5	13.9	9.4	11.9	15.9	29.8	9.1	6.1	7.9	10.5	19.4
Softeners, emollients (A06AA)														
0	92.7	93.4	94.2	95.1	96.2	96.8	96.6	96.1	93.2	97.3	97.9	97.6	97.3	94.7
1–2	5.7	5.2	4.6	3.9	3.1	2.9	2.8	2.9	5.0	2.2	2.0	2.0	2.2	4.2
3 or more	1.6	1.4	1.2	1.0	0.7	0.2	0.6	0.9	1.8	0.4	0.2	0.3	0.6	1.2
Contact laxatives (A06AB)														
0	72.9	73.4	74.1	74.7	75.0	73.5	73.7	75.1	85.4	75.9	74.3	74.7	76.0	86.0
1–2	26.9	26.4	25.8	25.3	24.9	26.4	26.2	24.9	14.5	24.1	25.6	25.3	24.0	13.9
3 or more	0.2	0.1	0.1	0.0	0.0	0.0	0.0	0.0	0.1	0.0	0.0	0.0	0.0	0.0
Bulk-forming laxatives (A06AC)														
0	85.0	86.4	88.1	89.6	91.7	92.8	91.9	91.4	88.2	93.6	94.5	93.7	93.3	91.8
1–2	10.7	9.9	8.8	7.9	6.7	6.5	6.9	6.1	8.0	5.5	5.1	5.7	5.3	6.3
3 or more	4.3	3.7	3.1	2.4	1.7	0.7	1.2	2.6	3.8	0.9	0.4	0.7	1.4	1.9
Osmotically acting laxatives (A06AD)														
0	17.1	18.7	20.2	22.0	24.4	28.0	22.4	22.6	29.8	28.5	31.5	26.4	26.6	35.9
1–2	58.3	60.0	61.4	62.9	64.5	65.0	68.0	64.8	45.8	64.5	64.0	67.8	65.1	48.3
3 or more	24.6	21.3	18.4	15.1	11.0	7.0	9.5	12.6	24.4	7.0	4.5	5.9	8.3	15.8
Enemas (A06AG)														
0	84.0	85.4	86.9	88.5	90.4	89.5	91.4	91.0	86.5	92.2	91.4	92.9	92.8	89.4
1–2	14.4	13.3	12.2	10.8	9.2	10.3	8.3	8.5	12.5	7.6	8.4	7.0	7.0	10.1
3 or more	1.6	1.3	1.0	0.7	0.4	0.3	0.2	0.5	1.0	0.2	0.2	0.1	0.2	0.5
Antidiarrheals, intestinal antiinflammatory/antiinfective agents (A07)														
0	51.2	55.7	60.6	67.0	75.0	67.6	77.7	78.5	71.7	82.3	77.1	84.4	85.0	77.7
1–2	31.6	30.0	27.8	24.5	19.6	27.4	17.6	16.0	19.1	14.7	20.2	12.9	11.9	16.2
3 or more	17.1	14.3	11.6	8.4	5.4	5.0	4.7	5.5	9.2	3.1	2.6	2.7	3.1	6.1
Antipropulsives (A07D)														
0	68.4	72.0	75.6	80.1	85.2	82.7	86.6	86.4	80.9	89.7	88.2	90.8	90.7	85.2
1–2	23.1	21.0	18.6	15.6	12.1	15.4	10.9	10.5	14.1	8.8	10.9	7.8	7.7	11.7
3 or more	8.5	7.0	5.7	4.2	2.7	1.9	2.5	3.1	5.0	1.5	1.0	1.4	1.6	3.1
Other antidiarrheals (A07X)														
0	69.7	72.9	76.5	81.0	86.3	80.6	88.0	89.4	84.7	90.7	86.9	91.8	92.8	88.6
1–2	24.6	22.4	20.0	16.8	12.5	18.3	11.0	9.5	13.3	8.8	12.5	7.7	6.6	10.3
3 or more	5.7	4.6	3.4	2.2	1.1	1.2	1.0	1.1	2.0	0.6	0.5	0.5	0.6	1.1
Antidepressants														
0 reimbursement	63.9	66.9	70.2	73.8	78.7	86.1	77.6	76.2	71.2	83.1	89.7	82.3	80.6	76.1
1–2	10.3	9.4	8.4	7.4	5.9	6.3	6.0	5.0	6.5	6.4	5.2	6.6	6.6	8.0
3 or more	25.8	23.7	21.5	18.8	15.4	7.6	16.3	18.7	22.3	10.5	5.2	11.0	12.8	15.8
Neuroleptics														
0	92.8	93.6	94.3	95.3	96.4	97.3	96.1	96.5	95.3	97.2	98.0	96.9	97.2	96.2
1–2	2.8	2.4	2.1	1.6	1.1	1.1	1.2	0.9	1.6	1.2	0.9	1.2	1.2	1.8
3 or more	4.4	4.0	3.6	3.1	2.5	1.7	2.7	2.6	3.2	1.6	1.1	1.9	1.6	2.0
Anxiolytics														
0	42.1	46.2	51.2	57.6	67.5	74.5	66.8	65.4	58.1	76.6	83.1	76.5	74.0	66.7
1–2	23.8	23.2	22.1	20.2	16.0	17.7	17.3	13.9	12.2	13.3	12.4	13.8	13.1	14.0
3 or more	34.1	30.6	26.7	22.3	16.4	7.9	15.9	20.6	29.7	10.1	4.5	9.6	12.8	19.3
Hypnotics														
0	72.1	74.8	78.0	81.5	86.0	93.5	86.0	82.1	77.8	89.7	95.6	90.0	86.2	82.6
1–2	12.2	11.1	9.6	8.1	6.0	4.1	6.8	6.5	5.7	5.4	2.8	5.5	7.0	7.3
3 or more	15.7	14.1	12.4	10.4	8.0	2.4	7.3	11.4	16.4	4.9	1.6	4.4	6.7	10.1

^a^excluding drugs indicated for purge in a context of colonoscopy preparation

*P < 0.0001 for distribution comparisons between “all −1 year” vs “all 1 year”

### Outpatient visits

During the year preceding hospitalisation, 96% of IBS patients, regardless of age, had seen their GP more than once and 84% had seen their GP at least 3 times, while 78% of patients had seen a gastroenterologist (only two-thirds of patients 75 years or older) (Table 6). Nevertheless, during the five years before hospitalisation, 82% of patients had seen a gastroenterologist at least once and 16% had seen a gastroenterologist at least three times. These frequencies decreased during the year after hospitalisation with 19% of patients with at least one visit and 3% of patients with at least three visits. At least three visits to specialists, including gastroenterologists, were identified for 37% of patients during the year before and 28% of patients during the year after hospitalisation. About 10% of patients consulted a psychiatrist at least once during the year before or after the index hospitalisation.

**Table 6:** Outpatient visits five years to one year before, or one year after hospitalisation of patients with a hospital diagnosis of IBS in 2015 in France

	Before hospitalisation									After hospitalisation				
Years	−5	−4	−3	−2	−1 year					+ 1 year				
	All	All	All	All	All*	18–39	40–59	60–74	75 +	All*	18–39	40–59	60–74	75 +
N					29,509	7040	12,081	7883	2505					
	%	%	%	%	%	%	%	%	%	%	%	%	%	%
General practitioner														
0	1.1	1.3	1.6	2.1	3.6	3.3	3.2	3.1	8.6	6.6	7.4	6.2	4.6	12.9
1–2	1.4	1.8	2.5	4.5	12.3	14.4	13.9	9.1	8.5	15.4	20.0	16.8	11.3	9.0
≥ 3	97.4	96.9	95.9	93.4	84.1°	82.4	83.0	87.8	82.9	77.9°	72.7	77.0	84.0	78.2
Gastroenterologist														
0	17.5	18.2	19.2	20.2	21.6	21.9	19.6	20.4	34.2	80.8	77.9	82.6	81.0	79.2
1–2	67.0	68.9	70.8	72.6	74.3	73.2	77.0	75.3	61.0	16.1	18.5	14.7	15.8	17.2
≥ 3	15.5	12.9	10.0	7.2	4.1	4.9	3.4	4.2	4.9	3.1	3.5	2.7	3.2	3.6
Psychiatrist														
0	78.0	80.4	82.9	86.1	90.0	91.2	89.1	90.3	90.1	89.1	89.8	88.0	90.1	89.1
1–2	9.8	8.8	7.7	6.4	4.9	4.3	4.7	5.1	7.3	5.3	4.9	5.3	5.1	7.5
≥ 3	12.3	10.9	9.3	7.5	5.1	4.5	6.2	4.7	2.7	5.6	5.3	6.7	4.8	3.4
All specialists														
0	2.3	2.7	3.4	4.4	6.1	6.5	5.3	5.6	10.6	42.7	40.8	45.2	42.7	36.6
1–2	26.1	30.3	36.1	43.9	56.5	54.3	58.5	57.2	50.2	29.7	31.2	28.2	29.8	31.7
≥ 3	71.6	67.0	60.6	51.7	37.4	39.2	36.2	37.2	39.2	27.6	28.0	26.5	27.5	31.7

*P < 0.0001 for distribution comparisons between “all −1 year” vs “all 1 year”)

## Discussion

Constitution of a large cohort of patients from SNDS data, studied 5 years before and one year after the index hospitalisation, allowed description of the high level of IBS-related healthcare use in France, comprising the use of various drugs, and numerous consultations and examinations.

This national observational study using the French reimbursement database included almost 30,000 patients with at least hospitalisation principal diagnosis ICD-10 code for IBS in 2015. This study is the largest study to be conducted in France in terms of the number of patients included and is also one of the largest studies to be published in literature. In France, only LTD codes are recorded for each healthcare use, while only primary or secondary diagnosis codes are recorded during a hospitalisation functional disorders like IBS. It must be noted the difficulty of confidently asserting IBS diagnosis for physicians in front of a patient with digestive symptoms, even for gastroenterologists after a normal colonoscopy [21]. However, underestimation of the prevalence of IBS has also been described in an English study based on codes for the reasons for general practice visits [22]. Patients included in our study presented similar sociodemographic characteristics, with a mean age of about 50 years and two-thirds of women, to those of other studies on IBS performed in France and in other countries [7, 23]. Although the proportion of patients with the various subtypes according to bowel habit cannot be clearly defined on the basis of ICD-10 codes, as there is no code for IBS-C or IBS-M, one-third of patients presented an IBS-D pattern, which is consistent with the results of other published large population-based studies [4, 24].

The primary objective of this study was to define healthcare consumption of IBS patients not only based on patient reports but on real reimbursement data. One strength of this study is that the use of SNDS database allowed healthcare consumption to be studied over a period of 7 years, 5 years before and one year after the hospitalisation in 2015, providing a dynamic perspective. Interestingly, 3% of these IBS patients had been previously hospitalised for IBS management during the 5 years before the index hospitalisation and 1% were hospitalised during the year after the index hospitalisation. Thirty percent of patients were admitted via the emergency room, a much higher percentage than the 2 to 5% usually reported in North America and Europe [3]. The most common reasons for hospitalisation during the five years before the index hospitalisation were also digestive disorders, particularly abdominal pain in 5% of cases. The severity of symptoms, particularly abdominal pain, has been previously described; in general practice in England, pain is the most common symptom one year before IBS diagnosis and, in the US, pain is the leading symptom in both the ambulatory setting and in the emergency room [12, 22]. In another study, the severity and duration of pain were the most important factors for healthcare seeking [22, 25]. During the five years before the index hospitalisations, patients had also been hospitalised for various digestive disorders other from IBS, which could represent differential diagnoses (colonic diverticulosis, appendicitis, gallbladder disorders), as previously reported [13, 26]. These hospitalisations could also reflect a high rate of comorbidities among IBS patients, as reported by other authors during 8-year follow-up of IBS patients compared to controls [27]. However, hospitalisations were less frequent during the year after the diagnosis of IBS.

Outpatient visits were frequent and concerned primary care physicians (GP) for almost all patients. About 80% of patients saw their GP more than 3 times a year before the diagnosis of IBS. A slight decrease in the number of repeated visits to the general practitioner was observed after the diagnosis of IBS, but the number of visits remained relatively constant over the 7-year follow-up period with more than 80% of patients having more than 3 GP visits per year. The number of GP visits may depend on the healthcare system and reimbursement rules, as the number of visits observed in this study is higher than that observed in less favourable reimbursement systems: only one-half of US patients visit a primary care physician each year with an average of two to three visits [3]. Patients also frequently consulted a gastroenterologist before the diagnosis (about 80% of patients during the previous years), but with fewer repeated visits. This percentage was higher than the 50% of gastroenterologist visits observed during the 2 years around the diagnosis of IBS in the USA [23]. However, in our study after hospitalisation and diagnosis of IBS, the number of visits decreased, especially gastroenterologist visits that fell to less than 25%. It should be noted that 14% of patients during the previous 5 years and 10% of patients during the year after hospitalisation consulted a psychiatrist, and about one third of patients took antidepressant or anxiolytics reflecting the well-known prevalence of anxiety and depression, at least in a subgroup of IBS patients, which also constitutes an important healthcare seeking factor in some studies [28–30].

This study identified the examinations performed as part of the management of IBS in France. The examination most commonly performed was colonoscopy, in more than two-thirds of patients, regardless of their age (below or above 50 years). This percentage is higher than the European average of 30% and the 45% rate observed in the USA, where it now represents a small proportion of indications for colonoscopy [3, 23, 31, 32]. One-third of patients also underwent upper GI endoscopy. Radiological examinations were performed in a large number of cases, especially before the diagnosis of IBS: abdominal ultrasound in 58% and CT scan in 25% of patients, but also sometimes after the diagnosis, reflecting the patients’ concern in the absence of reassurance even after a normal colonoscopy, but also the ease of medical nomadism in France compared to other countries such as Great Britain. However, similar percentages of radiological examinations were recently described in the US [23, 33]. The laboratory tests most commonly performed in 2015 (TSH, CRP, serum test for coeliac disease) mostly complied with the recommended assessment to eliminate differential diagnoses, particularly in the case of IBS-D or IBS-M, but they were repeated too frequently before or even after the diagnosis. This large number of examinations may also reflect the degree of confidence of the physician in the positive diagnosis or exclusion diagnosis of IBS, as commonly described for primary physicians and non-gastroenterologists [21].

Almost all patients received at least one drug prescription during the 7-year follow-up, either before or after the diagnosis of IBS. The treatments most commonly prescribed were laxatives, particularly osmotically acting laxatives and antispasmodics, with little change in these therapeutic classes after the diagnosis of IBS. This percentage of 95% laxative use is somewhat surprising, as one-third of ICD-10 codes corresponded to IBS-D. This high percentage could be explained by the frequent practice of general practitioners and even gastroenterologists to classify diarrhoea as “spurious diarrhoea” associated with constipation but mainly because in about half of them it corresponded to a purge in a context of colonoscopy preparation.

Societal cost may represent a large share of the disease burden. This patient population with IBS presented a large number of sick leaves, as 42% of patients between the ages of 18 and 39 years and 35% of patients between the ages of 40 and 59 years had at least one sick leave, although no information was available concerning the reason for sick leave. Similar percentages have also been reported in the UK [34].

### Strengths and limitations

The main strengths of this study concern the use of the SNDS population database comprising almost 84% of all drug dispensing data in 2015. This study provides an overview of healthcare consumption for IBS in France, but did not evaluate possible disparities between various regions of France, as recently reported in the USA [23]. Due to the nature of this study, healthcare use cannot be analysed according to disease severity, which constitutes a major predictive factor [35]. Some of the results of this first analysis of SNDS data must be interpreted cautiously, as people not included in this study and covered by other health insurance schemes could present different age structures and characteristics, exposures and healthcare use. Apart from the classical limitations of this type of claims database, certain events may have been underestimated, especially hospital radiological examinations and laboratory tests, which cannot be identified or OTC drugs or alternative therapies not present in the SNDS.

## Conclusions

This first study using SNDS to assess healthcare consumption in IBS highlights value of large claims databases including diseases to study healthcare use based on reimbursement data. This study reveals a number of healthcare expenditures that may not be necessarily warranted, such as the large number of repeated outpatient visits, examinations and particularly radiological examinations, and numerous and urgent hospitalisations, which can be partly explained by the difficult patient-healthcare provider relationship in IBS that can increases healthcare seeking. Altogether, these data encourage the publication of physician guidelines by national learned societies and the development of therapeutic education in IBS to ensure more cost-effective management.

## Data Availability

Datasets are localised at Cnam and access is authorized by the CNIL (French data protection authority). All data were encrypted using a unique identifier and all records used were anonymized.

## Abbreviations

ATC: Anatomical Therapeutic Classification
Cnam: *Caisse Nationale d’Assurance Maladie* general health scheme fund
CRP: C-reactive protein
GI: GastroIntestinal
IBS: Irritable bowel syndrome
ICD-10: International classification of diseases, 10th edition
LOS: length of hospital stay
LTD: long-term diseases
MRI: Magnetic Resonance Imaging
PMSI: Programme de médicalisation des systèmes d’information (PMSI) [Medical Information System Programme]
SNDS: Système National des données de santé [National Health data Information System]
TSH: Thyroid-Stimulating Hormone

## References

[1] Lacy Brian E., Mearin Fermín, Chang Lin, Chey William D., Lembo Anthony J., Simren Magnus, Spiller Robin (2016). Bowel Disorders. Gastroenterology.

[2] Ford AC, Lacy BE, Talley NJ (2017). Irritable bowel syndrome. N Engl J Med.

[3] Canavan C, West J, Card T (2014). The epidemiology of irritable bowel syndrome. Clin Epidemiol.

[4] Hungin APS, Whorwell PJ, Tack J, Mearin F (2003). The prevalence, patterns and impact of irritable bowel syndrome: an international survey of 40,000 subjects. Aliment Pharmacol Ther.

[5] Lovell RM, Ford AC (2012). Global prevalence of and risk factors for irritable bowel syndrome: a meta-analysis. Clin Gastroenterol Hepatol.

[6] Jung HK, Kim YH, Park JY, Jang BH, Park SY, Nam MH (2014). Estimating the burden of irritable bowel syndrome: analysis of a nationwide Korean database. J Neurogastroenterol Motil.

[7] Brun-Strang C, Dapoigny M, Lafuma A, Wainsten JP, Fagnani F (2007). Irritable bowel syndrome in France: quality of life, medical management, and costs: the Encoli study. Eur J Gastroenterol Hepatol.

[8] Schnabel L, Buscail C, Sabate JM, Bouchoucha M, Kesse-Guyot E, Allès B (2018). Association between ultra-processed food consumption and functional GI disorders: results from the French NutriNet-Santé cohort. Am J Gastroenterol.

[9] Canavan C, West J, Card T (2015). Change in quality of life for patients with irritable bowel syndrome following referral to a gastroenterologist: a cohort study. PLoS One.

[10] Halpert A, Dalton CB, Palsson O, Morris C, Hu Y, Bangdiwala S (2010). Irritable bowel syndrome patients’ ideal expectations and recent experiences with healthcare providers: a national survey. Dig Dis Sci.

[11] Halpert Albena (2018). Irritable Bowel Syndrome: Patient-Provider Interaction and Patient Education. Journal of Clinical Medicine.

[12] Peery AF, Crockett SD, Barritt AS, Dellon ES, Eluri S, Gangarosa LM (2015). Burden of GI, liver, and pancreatic diseases in the United States. Gastroenterology..

[13] Kennedy TM, Jones RH (2000). Epidemiology of cholecystectomy and irritable bowel syndrome in a UK population. Br J Surg.

[14] Poulsen CH, Eplov LF, Hjorthøj C, Hastrup LH, Eliasen M, Dantoft TM, et al. Irritable bowel symptoms, use of healthcare, costs, sickness and disability pension benefits: a long-term population-based study. Scand J Public Health. 2018. 10.1177/1403494818776168. 10.1177/1403494818776168 29762084

[15] Ford AC, Forman D, Bailey AG, Axon AT, Moayyedi P (2008). Irritable bowel syndrome: a 10-yr natural history of symptoms and factors that influence consultation behavior. Am J Gastroenterol.

[16] Guerin A, Carson RT, Lewis B, Yin D, Kaminsky M, Wu E (2014). The economic burden of treatment failure amongst patients with irritable bowel syndrome with constipation or chronic constipation: a retrospective analysis of a Medicaid population. J Med Econ.

[17] Buono JL, Mathur K, Averitt AJ, Andrae DA (2017). Economic burden of inadequate symptom control among US commercially insured patients with irritable bowel syndrome with diarrhea. J Med Econ.

[18] Cash B, Sullivan S, Barghout V (2005). Total costs of IBS: employer and managed care perspective. Am J Manag Care.

[19] Flik CE, Laan W, Smout AJ, Weusten BL, de Wit NJ (2015). Comparison of medical costs generated by IBS patients in primary and secondary care in the Netherlands. BMC Gastroenterol.

[20] Tuppin P, Rudant J, Constantinou P, Gastaldi-Ménager C, Rachas A, de Roquefeuil L (2017). Value of a national administrative database to guide public decisions: from the Système National d'Information Interrégimes de l'Assurance Maladie (SNIIRAM) to the Système National des Données de Santé (SNDS) in France. Rev Epidemiol Sante Publique.

[21] Andresen V, Whorwell P, Fortea J, Auzière S (2015). An exploration of the barriers to the confident diagnosis of irritable bowel syndrome: a survey among general practitioners, gastroenterologists and experts in five European countries. United Eur Gastroenterol J.

[22] Harkness EF, Grant L, O'Brien SJ, Chew-Graham CA, Thompson DG (2013). Using read codes to identify patients with irritable bowel syndrome in general practice: a database study. BMC Fam Pract.

[23] Lacy Brian E., Patel Haridarshan, Guérin Annie, Dea Katherine, Scopel Justin L., Alaghband Reza, Wu Eric Qiong, Mody Reema (2016). Variation in Care for Patients with Irritable Bowel Syndrome in the United States. PLOS ONE.

[24] Le Pluart D, Sabate J-M, Bouchoucha M, Hercberg S, Benamouzig R, Julia C (2015). Functional GI disorders in 35,447 adults and their association with body mass index. Aliment Pharmacol Ther.

[25] Talley NJ, Boyce PM, Jones M (1997). Predictors of health care seeking for irritable bowel syndrome: a population based study. Gut..

[26] Longstreth GF, Yao JF (2004). Irritable bowel syndrome and surgery: a multivariable analysis. Gastroenterology..

[27] Faresjö Å, Grodzinsky E, Hallert C, Timpka T (2013). Patients with irritable bowel syndrome are more burdened by co-morbidity and worry about serious diseases than healthy controls--eight years follow-up of IBS patients in primary care. BMC Public Health.

[28] Hillilä MT, Siivola MT, Färkkilä MA (2007). Comorbidity and use of health-care services among irritable bowel syndrome sufferers. Scand J Gastroenterol.

[29] Guthrie E, Creed F, Fernandes L, Ratcliffe J, Van Der Jagt J, Martin J (2003). Cluster analysis of symptoms and health seeking behaviour differentiates subgroups of patients with severe irritable bowel syndrome. Gut..

[30] Ringström G, Abrahamsson H, Strid H, Simrén M (2007). Why do subjects with irritable bowel syndrome seek health care for their symptoms?. Scand J Gastroenterol.

[31] Lieberman DA, Williams JL, Holub JL, Morris CD, Logan JR, Eisen GM (2014). Colonoscopy utilization and outcomes 2000 to 2011. Gastrointest Endosc.

[32] Soncini M, Stasi C, Usai Satta P, Milazzo G, Bianco M, Leandro G, et al M; AIGO. IBS clinical management in Italy: The AIGO survey. Dig Liver Dis. 2018 Oct 22. pii: S1590–8658(18)31200–3. 10.1016/j.dld.2018.10.006 30448159

[33] Spiegel BMR, Gralnek IM, Bolus R, Chang L, Dulai GS, Naliboff B (2005). Is a negative colonoscopy associated with reassurance or improved health-related quality of life in irritable bowel syndrome?. Gastrointest Endosc.

[34] Silk DB (2001). Impact of irritable bowel syndrome on personal relationships and working practices. Eur J Gastroenterol Hepatol.

[35] Drossman DA, Chang L, Bellamy N, Gallo-Torres HE, Lembo A, Mearin F (2011). Severity in irritable bowel syndrome: a Rome Foundation working team report. Am J Gastroenterol.

